# Effect of LED photobiomodulation on dental implant osseointegration: An in vivo study

**DOI:** 10.34172/joddd.2023.36954

**Published:** 2023-04-03

**Authors:** Jean-Marc Foletti, Floriane Remy, Luc Chevenement, Manon Sterba, Patrick Tavitian, Laurent Badih, Olivia Kenck-Veran

**Affiliations:** ^1^Department of Oral and maxillofacial Surgery, APHM, Conception University Hospital, Marseille, France; ^2^Aix-Marseille Univ, Gustave Eiffel Univ, LBA, Marseille, France; ^3^Glad Medical SAS, Salon-de-Provence, France; ^4^Department of Dentistry, APHM, la Timone University Hospital, Marseille, France; ^5^Dental Private Practice, Selestat, France

**Keywords:** Animal experiments, Bone implant interactions, Photobiomodulation, Histological studies, Dental implants

## Abstract

**Background.:**

Photobiomodulation (PBM) may be prescribed after dental surgery to accelerate tissue healing and improve implant stability. The objective of this study is to evaluate the efficiency of LED-PBM on the dental implant osseointegration.

**Methods.:**

A total of 48 implants (Kontact^TM^) were inserted in 8 Yucatan minipigs (6 implants per minipig) divided into 2 groups (test and control). The test group received LED-PBM with a total energy of 124.2 J/cm^2^ delivered over 4 sessions (at day0, day+8, day+15 and day+28) lasting 12 minutes each. At day+28, all animals were sacrificed, and their mandibles removed to perform histologic and histomorphometric analysis. Implant osseointegration was evaluated using the computation of bone/implant contact (BIC) index and bone surface/total surface (BS/ TS) ratio. The groups were compared using Student’s unpaired *t* test.

**Results.:**

BIC index and BS/TS ratio were significantly higher within the test group as compared to the control group (*P*<0.01). Histologic observations on bone tissues demonstrated that LED-PBM may improve and accelerate dental implant osseointegration: 25% of dental implants analyzed within the test group were completely osseointegrated, versus 12.5% within the control group.

**Conclusion.:**

This experimental study indicates that LED-PBM contributes to enhancing implant treatment outcomes.

## Introduction

Edentulism may be caused by several factors such as: diseases and social condition, influencing the history of oral and dental conditions.^1^ According to a recent study, even though the prevalence of complete edentulism has been reduced over the last decade, especially in developed countries, tooth loss remains a significant issue among the elderly population.^2^ Because tooth loss affects mastication, speech, esthetics and quality of life,^3^ it should be managed early on and as efficiently as possible. Dental implants are the most effective method for managing tooth loss, with success rates ranging from 90% to 95%.^4^

These success rates are strongly associated with the physicochemical properties of titanium: its surface oxide layer reacts, achieving a structural and functional connection with the surrounding soft tissue. This process is central to osseointegration.^5,6^ However, various factors may lead to inflammatory processes such as peri-implantitis or peri-implant mucositis that can lead to implant failure if not properly treated.^7^ These conditions affect the surrounding tissue of an implant and may result in loss of the supporting bone.^8^ Yet, according to recent studies, these conditions respectively affect between 1-47% and 19-65% of the population.^7,9,10^

Photobiomodulation (PBM), is a non-invasive irradiation procedure that dental clinicians may recommend to their patients in order to accelerate tissue healing and improve the stability of the implant.^11^ It consists in using a light within the red to near-infrared red wavelength in order to stimulate cellular activity in the peri-implant tissue.

The ATP38® medical device (Biotech Dental, Salon de Provence, France) performs PBM using light-emitting diodes (i.e., without thermal radiation) in order to treat large surfaces with the appropriate amount of energy. Its wavelengths range from 450 to 835 nm, which fits with the absorption peaks of cytochrome c oxidase and porphyrin (mitochondrial photoreceptors). Thus, this device aims to stimulate cell growth and the production of adenosine triphosphate (ATP).^12^

Our experimental study was designed in order to evaluate the *in vivo* effects of LED-PBM as performed by the ATP38® device regarding dental implant osseointegration.

## Materials and Methods

 The experimental study was performed on eight 18-month-old male Yucatan minipigs within a certified experimental facility (CERC, Aix Marseille Univ, Marseille France – agreement number: B1305522).

###  Study sample

This study intended to evaluate the effects of LED-PBM on dental implant osseointegration. The hypothesis was that LED-PBM contributes to enhancing implant osseointegration. Eight animals with comparable sizes, ages and weights were received from the same breeder. After a veterinary checkup that confirmed that all animals were in similar general good health, they were randomly assigned to test group (four animals) and control group (four animals) using a simple randomization technique. The test group received four sessions of PBM treatment on D0, D8, D15 and D28 after surgery. The animals in the control group did not receive PBM treatment after surgery. The treatment plan was chosen to ensure four sessions, uniformly distributed during the early bone healing stages for this experimental model.^13^

The sample size was determined on the basis of previous studies highlighting a 10% difference in BIC index depending on whether or not the subjects received PBM treatment with a theoretical variance of 155 mm^2^. ^14–17^ We computed that to have a statistical power of 80 per cent, a minimum of 24 implants per group (experimental and control groups) was necessary, which required 8 animals (6 implants per animal).

###  Surgical protocol

 The surgical protocol consisted of two steps: extraction surgery followed by implantation surgery after an 8-week healing period.

 Housing and all procedures were performed within the CERC (Centre d’Enseignement et de Recherche Chirurgical) at the Faculty of Medicine in Marseille, France. The delivery of the animals was scheduled for at least 48 hours before the start of the experiment to allow time for them to acclimatize to the new environment. They were examined at the end of the acclimatization period, and only animals in good health were chosen for the experiment. The animals were kept in individual boxes (temperature: 25°C) with free access to water, and fed according to their weight. Feeding was suspended on the day before the operation. Prior to each surgical intervention, the Yucatan minipigs were fasted overnight in order to prevent vomiting. On the day of the surgery, they were administered pre-anesthetic medication including an intra-muscular injection of Stresnil (Elanco, France, 6 cc) and Zoletil 100 (Virbac, France – 8 cc). The Yucatan minipigs were placed on the surgical table in the supine position, received orotracheal intubation and were maintained under general anesthesia with a mixture of sufentanil (Vidal, France – 2 mg at 2 mg/h) and propofol (Vidal, France, 10 mL at 10 mL/h). Animal safety during the procedure was assessed through continuous monitoring of heart rate, breathing frequency, oxygen saturation and body temperature, with the assistance of a certified veterinarian.

**Figure 1:**
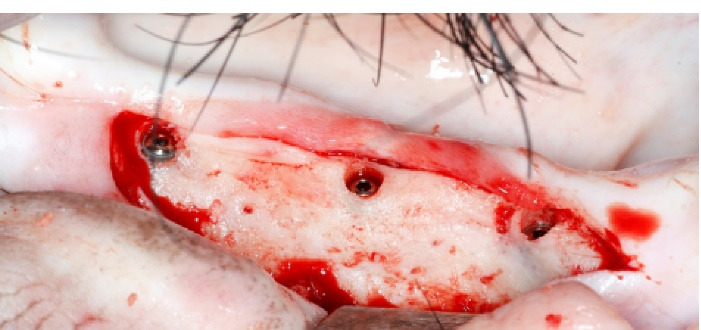
Three implants (121, 122 and 123) in place at D0 (right hemi-mandible), before mucosa closing.

For each animal, four mandibular premolars and the mandibular first molar were extracted under general anesthesia. After tooth extraction, the mucosa was stitched with VICRYL 3/0 (polyglactin 910, Ethicon, USA). Post-operative care included adequate diet consisting of soft foods, prophylactic antibiotic treatment (amoxicillin/clavulanic acid, Vidal, France – 1 g twice a day), and multi-day analgesia for 7 days. After an 8-week healing period, each Yucatan minipig received 6 implants (Kontact^TM^, Ø 4.2 mm, 10 mm length, Biotech Dental), 3 on each side of his mandible (Figure 1). Following an intra and extra-oral disinfection protocol, the mucoperiosteal flaps were elevated to access the expected implantation sites. Drilling was performed at the site of the previous incisions. In keeping with the manufacturer’s recommendations, progressive drilling was performed in 4 steps of increasing diameter, with continuous irrigation (drilling at 1500 rpm with 1.5- and 2-mm diameter drills followed by drilling at 1200 rpm with 3.2- and 4.2-mm diameter drills).

 The implant axis was checked at each step. For each implant, a minimum torque of 35 N.cm was achieved, using a cover screw. Finally, the mucosa was stitched with FLEXOCRIN 4/0 (copolyamide) (B. Braun, Germany). Implant placement was checked on retro alveolar X-rays, with an alignment key.

**Figure 2:**
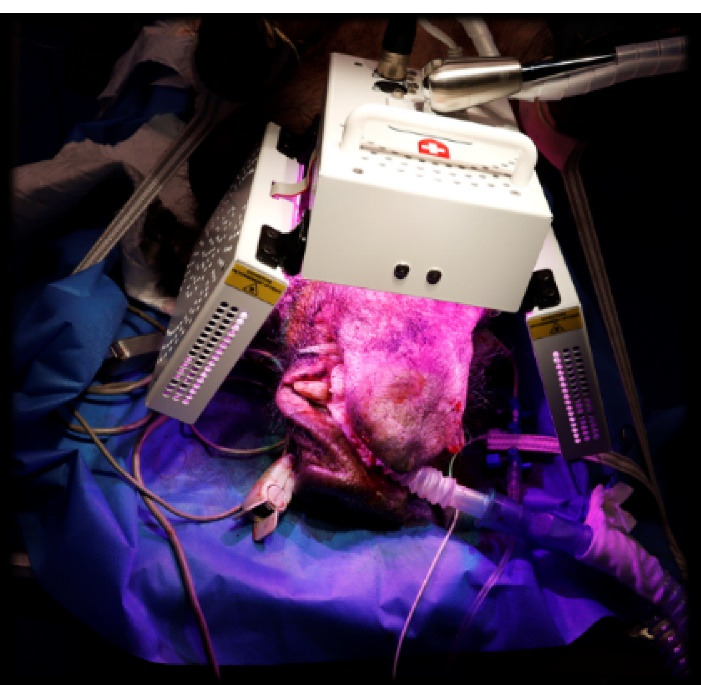
Set-up of the ATP38® medical device (Biotech Dental, France) used to perform the LED-photobiomodulation treatment on the experimental group animals: the three panels were placed at a 4 cm distance from the minipig cheeks (lateral panels) and lips (frontal panel).

Following the implantation surgery, the four Yucatan minipigs included in the test group underwent 4 sessions of PBM treatment delivered by the APT38® medical device on D0, D8, D15 and D28. This device consisted of a multi-panel system emitting cold polychromatic lights with a combination of wavelengths ranging from 450 to 835 nm in order to achieve healing, anti-inflammatory, and analgesic effects. For each session, the PBM treatment was applied with three panels of the device at a distance of 4 cm from the minipig’scheek (lateral panels) and lip (frontal panel) (Figure 2). Each PBM treatment involved the application of the 12-minute “analgesic, anti-inflammatory and healing” protocol defined for the ATP38® medical device. This protocol consisted of three irradiation steps leading to a total duration of 12 minutes and 10 seconds and a total of 124.2 J/cm^2^ administered. PBM parameters for each step are described in Table 1.

**Table 1:** Photobiomodulation parameters according to the “analgesic, anti-inflammatory and healing” protocol of the ATP38® medical device

	**Cold lights combination**					
	**Blue**	**Green**	**Amber**	**Red**	**Deep red**	**Infrared**
Wavelengths (nm)	470	525	590	620	680-760	820
Duration (s)	151/151/149	182/182/180	241/241/239	244/244/242	162/162/242	219/219/217
Fluency (J/cm^2^)	2/2/2	1/1/1	0.8/0.8 /0.8	2/2/2	4/4/4	4/4/4
Frequency (Hz)	70/5/0^a^					
Spot dimension area	600 cm^b^					
Distance from the target	4 cm					
Total energy delivery per session^c^	124.2 J/cm^2^					

Note:When necessary specific values are given for each step (1 to 3). ^a^ A null frequency (0 Hz) means continuous irradiation. ^b^ The spot dimension is calculated as the spot dimension of each panel (10 cm × 20 cm) multiplied by the number of used panel (3) ^c^ The total energy density was computed as the total fluency by panel and by step was 13.8 (J/cm^2^) multiplied by the number of step and the number of panel (3).

 Clinical and radiological controls were also performed on D0, D8, D15 and D28 under adequate sedation and mucosal fragments were collected for histological analyses.

**Figure 3:**
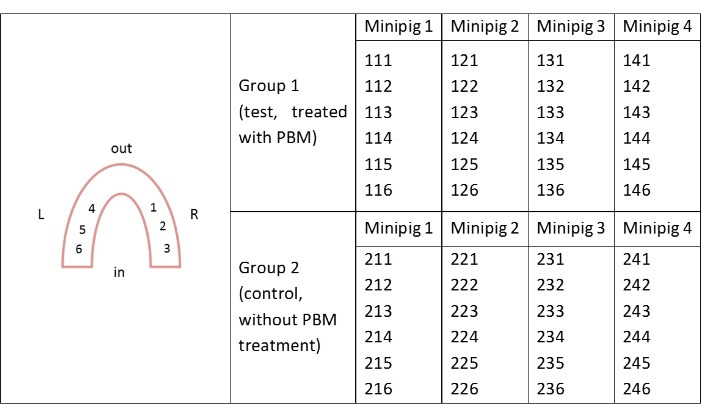
Implants numbering.

**Figure 4:**
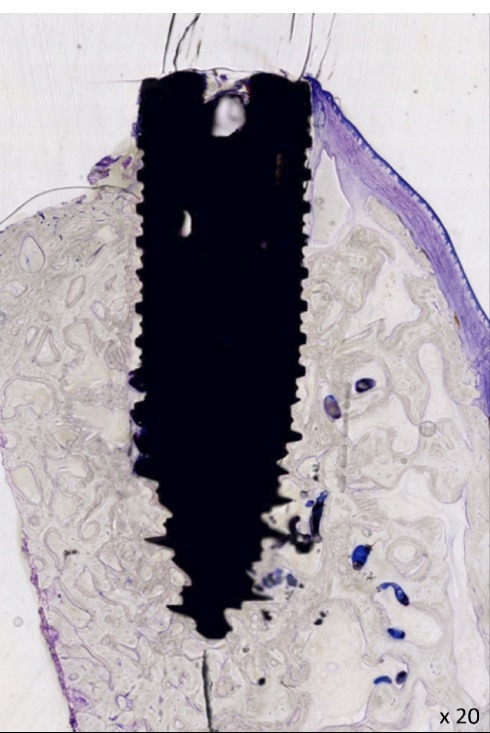
Histological illustration ( × 20), representation of the osseointegrated implant n°116 (D28). Soft tissue appears in blue/fuchsia and bone in grey.

On D28, all Yucatan minipigs were sacrificed by intra-muscular injection of a pentobarbital solution (Doléthal, Vetoquinol, France - 340 mL). The mandibles were collected ad integrum and immediately fixed in 4% formalin and sent to an external laboratory for sample preparation. The analyses were carried out by experts blinded to the assignment. The implants (n = 48) were isolated with surrounding bone tissues and identified by a unique number (Figure 3). All the samples were processed for non-decalcified histologic and histomorphometric analyses. These analyses were performed after a one-month dehydration period of the samples in a graded series of ethanol solutions (from 70 to 100%) impregnated with methyl methacrylate (Technovit 7200 VLC, Heraeus Kulzer, Germany). The samples were finally embedded in resin for polymerization under UV light. A radical initiator was added. After resin hardening, each implant was sectioned longitudinally down the middle with a diamond circular saw (Leica SP1600, Germany). These histological sections were glued onto a plastic slide and grinded with a polisher (Buelher Metaserv 2000). A half-block was then mounted onto a glass slide and serial sections of 30 μm thickness were generated using a diamond circular saw. McNeal’s (toluidine blue/basic fuchsine) staining of these histological sections was obtained according to standard operating procedures. Finally, all histological sections were digitized using a high-resolution Scanner (NanoZoomer 2.0; Hamamatsu Photonics) in bright field conditions with the objective × 20, on the MicroPICell platform (IRS, Nantes University, France) (Figure 4).

**Figure 5:**
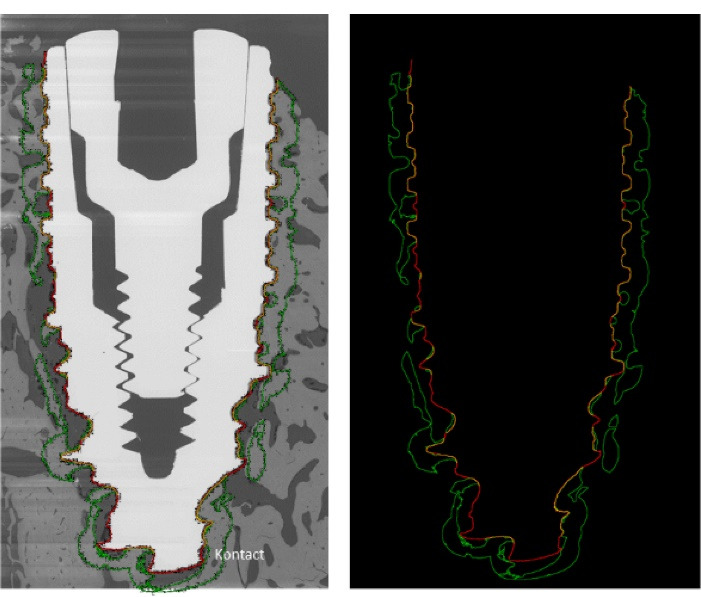
Example of BSEM image and its analyze with ImageJ software. The red line shows the dental implant edges, the yellow line shows the bone/implant contact, the green line delineates the bone at a 0.5 mm distance around the implant.

Each remaining second half-block was used to perform back-scattered electron microscopy (BSEM; Tabletop TM3000, Hitachi) to quantify bone-implant contact (BIC index) and bone surface to total surface ratio (BS/TS) at a distance of 0.5 mm around the implant. Contiguous images of the implant and the surrounding bone tissue were obtained with a magnification of × 50 and a motorized, programmable stage (Debel). On these BSEM images, the titanium implants appeared in white/light grey, the mineralized bone in grey and the non-mineralized tissue in black (Figure 5). BIC and BS/TS histomorphometric measurements were performed using ImageJ software (Figure 5).

###  Statistical analyses

All data (BIC; BS/TS 0.5 mm) were summarized/presented in mean ± standard-deviation. Results were interpreted by a blinded observer. The results obtained from the test and control groups were compared thanks to an unpaired student’s *t* test performed using BiostaTGV online statistical software (https://biostatgv.sentiweb.fr); the level of significance was set at 0.05. More specifically, the BIC and BS/TS histomorphometric measurements were compared, as well as the level of osseointegration and whether or not fibrointegration or inflammation were observed.

## Results

 48 implants (4.2 × 10 mm) were placed into 8 Yucatan minipigs’ mandibles. A 100 % global survival rate was observed within 28 days, prior to euthanasia. All animals survived, with a normal food intake during the course of our study. No peri-operative or post-operative complications were reported.

 Four implants (8,33%) were not suitable for histomorphometric measurements due to technical issues. These histological sections could not be analyzed either because they had been too damaged by sanding, or because the scanning machine could not be used for them (subject looked out of focus or blurry).

###  Bone tissue analyzes

Statistical analysis demonstrated a higher BIC and BS/TS at 0.5 mm in the test group when compared to the control group (*P* < 0.01). The mean BIC values were 48.1 ± 16% at 4 weeks in the test group and 29.6 ± 21% in the control group (*P* = 0.00094) (Table 2). The mean BS/TS values were 52.7 ± 16% in the test group and 34.5 ± 24% in the control group (*P* = 0.0023). The BIC and BS/TS at 0.5 mm values are summarized in Table 3.

**Table 2:** Histomorphometry global results. BIC and BS/TS values were significantly higher in the ATP38 test group than in the control group (*P* < 0.01 in both cases)

	**Test group**	**Control group**
BIC		
Mean	48,1%	29,6%
SD	16%	21%
Min	6%	0
Max	71%	66%
*P* value	0.00094	
BS/TS 0.5 mm		
Mean	53%	34%
SD	16%	24%
Min	13%	0
Max	75%	73%
*P* value	0.0023	

**Table 3:** BIC and BS/TS values (in %) for each implant, when available

**Test group**			**Control group**		
**Implant No.**	**BIC (%)**	**BS/TS (%)**	**Implant No.**	**BIC (%)**	**BS/TS (%)**
111	57	65	211	12	12
112	66	40	212	58	66
113	68	75	213	66	73
114	ND	ND	214	38	41
115	44	63	215	0	0
116	54	57	216	10	32
121	27	32	221	40	46
122	24	31	222	42	45
123	6	13	223	17	17
124	53	46	224	0	0
125	35	37	225	0	5
126	42	53	226	ND	ND
131	51	57	231	29	29
132	54	62	232	38	44
133	ND	ND	233	17	14
134	71	71	234	12	17
135	53	66	235	0	0
136	60	66	236	35	26
141	46	49	241	60	70
142	49	55	242	47	70
143	62	71	243	53	66
144	ND	ND	244	47	43
145	37	45	245	22	33
146	51	53	246	39	44

ND, non-disclosed.

 Observations of the histological sections stained with Mc Neal (toluidine blue/basic fuchsine) indicated that:

- Complete osseointegration was more likely to be achieved in the test group (6/24 implants; 25%) than in the control group (3/24 implants; 12, 5%).
- Fibrointegration and inflammation were more frequently encountered in the control group: 9 (37, 5%) implants in the control group were partially surrounded by non-calcified fibrous tissue (211, 215, 216, 223, 224, 225, 233, and 235) against only 1 (8%) implant (134) in the test group.

## Discussion

This experimental study on Yucatan minipigs aimed to assess whether LED-PBM is efficient in the enhancement of dental implant osseointegration. Several studies were carried out to assess the effect of light therapy on the osseointegration of dental implants^18^ and orthodontics mini-implants^11^ on animals and humans. However, the potential positive effect of PBM on the osseointegration of dental implants is still under debate. This lack of consensus could be explained by several factors including the absence of a unique PBM protocol and the study design inhomogeneity, in particular the choice of the experimental model.^11,18^ Among the main differences between PBM protocols is the kind of light sources used, which could be lasers (PBM/LLLT) or light emitting diodes (PBM/LED) as used in this study. Although the use of LED for PBM is more recent, it presents some advantages, such as a low price and fewer safety considerations compared to the use of laser.^19^ Considering the differences between these two light sources and the debates on whether these differences may affect treatment efficacy,^19^ additional data on the effect of LED-PBM are necessary.

Pre-clinical studies on animals enable BIC and BS/TS at 0.5 mm measurements on bone samples that may be more sensitive than the resonance frequency analysis mostly used to assess implant osseointegration in humans and then provide complementary data to clinical ones. While most of the animals studies on PBM were carried out on small animals, such as rats and rabbits,^11,18,20^ the present study involved minipigs that present similar bone properties to those of humans, enabled the study of implants with equivalent sizes as those used in clinical practice, and have a higher survival time.^21^

The results of the histomorphometric analyzes have demonstrated that BIC and BS/TS values were significantly higher in the ATP38 test group than in the control group (*P* < 0.001 in both cases), indicating an accelerated osseointegration in the LED/PBM group compared to the control one.

In both groups, BIC and BS/TS results at 4 weeks were in the same order of magnitude as those obtained in previous studies employing similar methodology.^22^ Furthermore, complete osseointegration was more frequently observed for implants in the ATP38 test group (55%) than for those in the control group (14%) 4 weeks after implant placement surgery. Both these histomorphometric and histological results point to an overall better osseointegration of the implants in the ATP38 test group. One could therefore hypothesize that osseointegration may be accelerated by the use of the ATP38.

A complete osseointegration was associated with high values of BIC and BS/TS, in the absence of clinical signs of tissue inflammation or fibrous tissue observed in contact with the implant. Observations of non-calcified fibrous tissue around some implants may be interpreted in two ways: it indicates an ongoing osseointegration, reflecting a local inflammatory physiological process for 5 implants, but means peri-implantitis or implant failure for 4 implants.^23^ In our study, inflammatory reactions were observed more frequently in the tissues surrounding the implants in the control group (19%) than around the implants in the test group (8%).

In their in vitro study, Rech et al^24^ showed that both PBM/LED and PBM/LLLT enhanced the cellular functions linked to peri-implant healing. In addition, Gulati et al^25^ showed a beneficial effect of LED/PBM in the prevention of crestal bone resorption. Our study corroborates these results by demonstrating a significantly higher bone osseointegration in the treated group. In a review published in 2021, Choe et al^26^ also highlighted the benefits of PBM, including LED-PBM and Laser-PBM, as a non-invasive photochemistry-based therapeutic approach capable of modulating inflammatory responses and reducing bacterial load after implant surgeries, thereby accelerating osseointegration. On the contrary, Bozkaya et al^27^ did not demonstrate a clinically significant effect of LLLT/PBM on implant stabilization in the early stages of alveolar bone healing. However, they studied the impact of laser application on dental implants. Furthermore, in Bozkaya and colleagues’ study, implant stability was measured by resonance frequency analysis, which may have a lower sensitivity than BIC and BS/TS assessments.

 Several methodological limitations should be addressed. Due to their young age, some Yucatan minipigs still had impacted teeth, which resulted in a traumatic extraction. Furthermore, despite the use of antibiotic prophylaxis and the feeding of suitable food textures, food deposits were observed next to the operated areas, which may account for local inflammation. However, this may not impact our comparative results given the similarities in the two groups, which shared the same diet, and probably had the same dental hygiene.

Some specific technical constraints prevented us from analyzing all inserted implants. As stated above, no histomorphometric analysis could be performed on 4 out of 48 implants (8.3%). One implant from the test group (144) was inserted too close to the adjacent canine. After radiographic control, it was left in place, as there was not enough space in order to accommodate a more distal implant. Nevertheless, no analysis was carried out on this implant. For two implants from the test group and one from the control group, the histological sections obtained were of insufficient quality. The instrumental techniques used in the preparation of half blocks, including cutting using a diamond saw and polishing, could be partially responsible for this failure. The cutting operation entails vibration levels and a 300 µm thickness loss approximately. To which can be added the challenge of homogeneously polishing two materials with substantially different hardnesses such as titanium and resin. This issue could be overcome by using laser cutting of soft tissue / implant block (Tissue Surgeon LLS Rowiak, Germany). This technique has already been experimented by Hoornaert et al.^22^ The methodology used in histomorphometric measurements (consisting in half-block cutting, use of electron microscopy in image acquisition and semi-automated digital image processing) has proved its efficiency in the literature. In order to measure BIC and avoid potential alteration of the samples, the implants and their adjacent tissues could be maintained *ad integrum.* Furthermore, the use of more conservative measurement methods, resonance frequency analysis or more recently, quantitative ultrasound techniques developed by Vayron et al could be relevant as well.^28^

Although PBM’s significant benefits regarding bone and mucosal healing have already been demonstrated in implant dentistry, no standard protocol for light irradiation has yet been defined. PBM’s multifaceted characteristics affect the comparability of studies. Variations in PBM’s parameters such as wavelength, radiant exposure or energy density (J/cm^2^), irradiance or power density (mW/cm^2^), exposure time, and delivery rate, have a varying impact depending on the targeted tissue and the intended therapeutic effect. PBM’s wavelength as reported in the literature varies between 600 and 1100 nm. The device used in our study (APT38) emits different wavelengths (ranging from 450 to 835 nm). The assessment of different protocols is outside of the scope of this study, that however demonstrates the therapeutic benefits of the evaluated protocol on both mucosal and bone healing in implant dentistry.

To gain more insight into the beneficial effect of this treatment, future studies must be carried out on the healing of the periodontal tissues. First of all, a preclinical study on minipig models including the measurement of the mucosal height, epithelium length and epithelium to platform distance as realized in Susin et al^29^ could be relevant to determine if the PBM has a significant effect on soft tissue healing. In addition, a multicentric clinical analysis that evaluate bone and soft tissue healing might be valuable to confirm the overall beneficial effect of this treatment in clinical practice.

## Conclusion

 This preclinical animal study was meant to assess the efficiency of PBM on the osseointegration of dental implants. Despite the above-mentioned limitations in this study, our results point to a significant increase in BIC and BS/TS at 0.5 mm in all Yucatan minipigs having benefitted from LED-PBM treatment with the ATP38® device.

 Considering these results, we assessed that LED-PBM performed using the ATP38® device contributes to enhancing implant treatment outcomes.

## Acknowledgments

 Special thanks are addressed to the Pr. S. Berdah, Responsible of the CERC (Centre d’enseignement et de recherche chirurgical, Aix Marseille Univ, Marseille France) who contributed to the experimental study by providing surgical and housing facilities.

## Competing Interests

 The authors declare that they have no conflicts of interest in relation to this article.

## Ethical Approval

 In accordance with the European Guidelines for Animal Care directive 2010/63/EU, ethical approval for animal experimentation was obtained from our local ethical committee (authorization no. APAFIS#23384-2019121920001912).

## Funding

 This study was funded by Glad Medical SAS.
